# Therapy-induced immunoediting and the evolution of cancer immune escape

**DOI:** 10.3389/fimmu.2026.1936590

**Published:** 2026-09-04

**Authors:** Shuang Meng, Zibo Wang

**Affiliations:** Qingdao Hospital, University of Health and Rehabilitation Sciences (Qingdao Municipal Hospital), Qingdao, Shandong, China

**Keywords:** cancer immunoediting, immune escape, longitudinal biomarkers, residual disease, therapy resistance

## Abstract

Anticancer therapy can eliminate immune-visible tumour cells while simultaneously imposing selective pressures that favour residual immune-evasive populations. This mini-review conceptualizes therapy-induced immunoediting as a dynamic continuum comprising pre-existing escape, therapeutic immune activation, a selective bottleneck, residual equilibrium and secondary escape. We discuss how genetic alterations, therapy-resistant stem-like tumour-cell states and spatial remodelling of vascular, stromal and myeloid niches cooperate to reduce tumour visibility, effector susceptibility and immune-cell access. Representative approved and investigational therapies illustrate both successful pharmacological interception and the limitations of non-selective immune intensification. We further highlight residual equilibrium as a clinically actionable window that may be identified through longitudinal tissue sampling, circulating tumour DNA and spatial or single-cell profiling. Clinically, longitudinal biomarkers may identify residual equilibrium and guide mechanism-matched treatment adaptation before immune-evasive populations expand into radiographically evident relapse.

## 1 Introduction

Cancer immunoediting describes the bidirectional interaction between malignant cells and host immunity through the sequential phases of elimination, equilibrium and escape (1–3). During elimination, innate and adaptive immune responses recognize and remove immunogenic tumour cells. Tumour variants that survive this pressure may enter a period of equilibrium, in which immune control restrains net growth while continuously selecting for less immunogenic or more immune-resistant subclones. Eventually, these selected populations can acquire sufficient capacity to evade immune recognition or destruction and progress into clinically apparent disease (4, 5). This framework has substantially advanced our understanding of tumour development by showing that immunity does not merely suppress cancer but also shapes its antigenic, molecular and cellular composition. Importantly, immunoediting can re-emerge during treatment, when therapeutic pressure creates a new round of tumour selection and adaptation (6, 7).

Modern anticancer therapies impose distinct but overlapping forms of immune selection. Immune checkpoint blockade restores or amplifies pre-existing antitumour T-cell activity, thereby preferentially eliminating immune-visible tumour cells (8, 9). Chemotherapy and radiotherapy can enhance antigen release, inflammatory signalling and immunogenic cell death, but may simultaneously induce tissue repair programmes, myeloid recruitment and stromal remodelling (10–12). Targeted therapies alter oncogenic signalling, differentiation states and cytokine production, whereas adoptive cell therapies impose intense pressure on tumour cells expressing defined target antigens (13, 14). Although these approaches differ mechanistically, incomplete tumour elimination may leave behind cell populations that are less visible to the immune system, less susceptible to immune-mediated killing or physically protected within suppressive tissue niches. Therapy can therefore both expose tumour vulnerabilities and create an evolutionary bottleneck from which immune-evasive populations emerge. Meanwhile, treatment-related fatigue, skeletal muscle loss and physical deconditioning may reduce physiological reserve and compromise treatment delivery, indicating that the evolution of tumour escape should also be considered alongside changes in host functional capacity (4, 15).

Current classifications distinguish primary, adaptive and acquired resistance and separately classify mechanisms as tumour-intrinsic or tumour-extrinsic (16). Primary resistance is present before treatment and may reflect low tumour antigenicity, defective antigen presentation or pre-existing immune exclusion. Adaptive resistance develops during treatment as tumour cells and the microenvironment respond to immune pressure, for example through inducible checkpoint expression, reversible antigen suppression or lineage plasticity. Acquired resistance emerges after an initial response and may involve selection of antigen-negative clones or stable defects in B2M or JAK–STAT signalling. These temporal categories intersect with the mechanistic classification: tumour-intrinsic mechanisms arise within malignant cells, whereas tumour-extrinsic mechanisms are mediated by immune, stromal, vascular or metabolic components of the microenvironment. Within our continuum, primary resistance corresponds mainly to pre-existing escape, adaptive resistance to therapeutic immune activation and the selective bottleneck, and acquired resistance to progression from residual equilibrium towards secondary escape, although these categories may overlap (17). Moreover, resistance is rarely driven by a single stable alteration. Genetic selection affecting tumour antigens, HLA expression, antigen-processing machinery or interferon signalling can coexist with reversible epigenetic states, lineage plasticity, cellular quiescence and spatial protection by stromal, vascular or myeloid compartments. Consequently, clinically apparent escape should be viewed as the outcome of a dynamic ecological and evolutionary process rather than as a fixed endpoint (18, 19).

**Figure 1:**
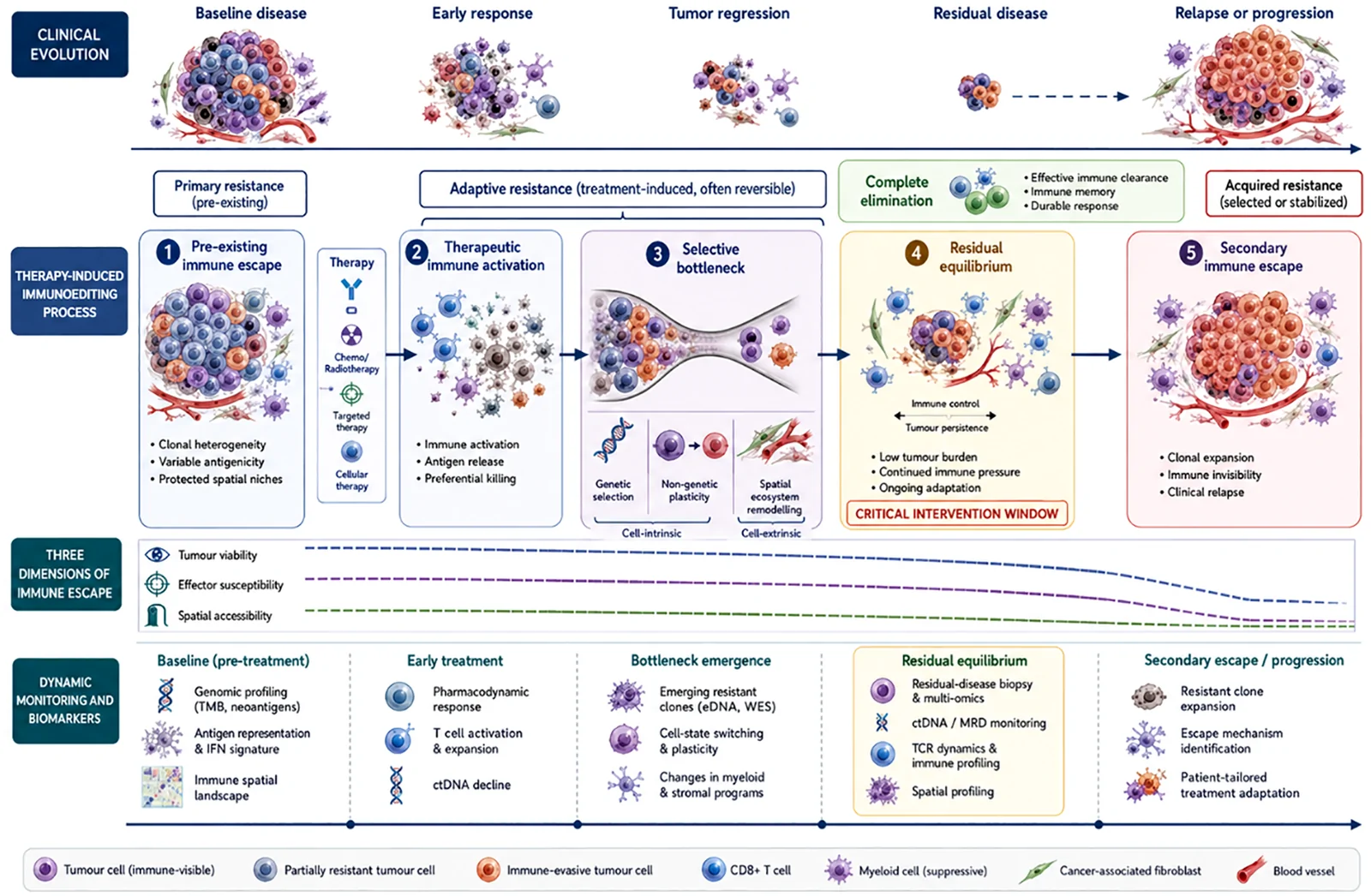
The therapy-induced immunoediting continuum. Anticancer therapy reshapes tumour evolution from baseline disease to early response, tumour regression, residual disease and potential relapse. Incomplete eradication creates a selective bottleneck driven by cell-intrinsic genetic selection and non-genetic plasticity together with cell-extrinsic spatial ecosystem remodelling. Surviving cells may enter residual equilibrium before evolving into secondary immune escape. Longitudinal genomic profiling, ctDNA, TCR dynamics and spatial immune analysis can help track these transitions and guide stage-matched intervention.

In this mini-review, we define “therapy-induced immunoediting” as the treatment-dependent selection, generation or stabilization of tumour and microenvironmental states that progressively reduce immune recognition, access or effector susceptibility. We propose a continuum comprising pre-existing immune escape, therapeutic immune activation, a selective bottleneck, residual equilibrium and secondary immune escape. Within this framework, we examine how genetic evolution, non-genetic plasticity and spatial ecosystem remodelling cooperate during treatment, and discuss how longitudinal preclinical models, dynamic biomarkers and stage-matched interventions may identify and disrupt escape before overt clinical progression. **Figure 1** provides an overview of this continuum by linking clinical tumour regression and relapse with evolving mechanisms of immune escape and longitudinal biomarker assessment. Viewing resistance through this temporal framework may help move the field from retrospective description of established resistance towards earlier detection and interception of evolving immune escape.

## 2 Therapy creates a new immunoediting landscape

Anticancer therapy does not act on a biologically uniform tumour (20). Instead, it is imposed on a heterogeneous population of malignant cells embedded within spatially distinct immune, stromal and vascular niches. Treatment therefore functions not only as a cytotoxic or immunostimulatory intervention but also as a selective force that changes the relative fitness of tumour subclones and cellular states (21). Sensitive populations are preferentially eliminated, whereas cells with reduced immunogenicity, impaired effector susceptibility or access to protective microenvironments may survive. This process can narrow tumour diversity during the initial response while simultaneously enriching residual populations with features that support subsequent immune escape. In this sense, therapy creates a new immunoediting landscape in which tumour evolution is shaped by both direct drug pressure and the immune responses mobilized by treatment.

Immune checkpoint inhibitors provide a clinically documented example of therapy-associated immunoediting. Pembrolizumab and nivolumab can restore antitumour T-cell activity, but the resulting immune pressure may selectively favour tumour populations that no longer respond to interferon signalling or present recognizable antigens (22). In paired tumour samples from patients with melanoma who initially responded to PD-1 blockade and subsequently relapsed, acquired loss-of-function alterations in JAK1 or JAK2 impaired interferon-receptor signalling, whereas B2M loss disrupted MHC class I antigen presentation (23). These observations provide direct evidence that an effective pharmacological intervention can create a selective bottleneck from which immune-evasive variants emerge. Importantly, such alterations are not universal mechanisms of resistance, but they illustrate how longitudinal sampling can distinguish treatment-associated clonal selection from baseline non-response (24).

Chemotherapy and radiotherapy impose broader and more complex forms of selection. Both can enhance tumour antigen release, damage-associated signalling and innate immune activation, thereby increasing immune recognition (25). However, their effects depend on dose, schedule, tissue context and the capacity of the host immune system to respond (26). Sublethal treatment may enrich stress-tolerant or apoptosis-resistant cells, while tissue injury can activate wound-healing programmes involving macrophages, fibroblasts, endothelial cells and extracellular matrix deposition (12). These responses may initially support antigen presentation and immune recruitment but can subsequently generate hypoxic, fibrotic or myeloid-dominant niches that protect residual tumour cells (18). Therefore, the same therapy can promote immune activation in one phase and facilitate immune escape in another.

Targeted therapies generate a different but overlapping evolutionary bottleneck by altering oncogenic signalling, differentiation programmes and tumour–immune communication. Initial pathway inhibition may increase antigen presentation or inflammatory chemokine production, whereas sustained pressure can select for pathway reactivation, bypass signalling, phenotypic switching or lineage plasticity (27, 28). These adaptive states may also reduce tumour visibility or change the immune composition of the microenvironment. Adoptive cellular therapies impose an even more focused pressure because tumour recognition often depends on one or a limited number of antigens. Deep responses can therefore be followed by antigen-low or antigen-negative relapse, altered lineage identity, impaired target accessibility or progressive effector-cell dysfunction (29).

Despite their mechanistic differences, these treatments converge on three determinants of immune escape: tumour visibility, susceptibility to immune killing and spatial accessibility to immune effectors. Therapy-induced immunoediting arises when incomplete treatment selectively preserves cells that are less visible, harder to eliminate or better protected within the tumour ecosystem. Understanding how each therapeutic modality reshapes these three dimensions is essential for identifying when an effective antitumour response begins to transition into residual equilibrium and eventual secondary escape (17).

## 3 Mechanisms driving escape across the treatment continuum

Therapy-induced immune escape rarely results from a single molecular event. Instead, genetic selection, reversible cell-state changes and spatial ecosystem remodelling can operate at different stages of treatment and reinforce one another (13). Pre-existing resistant subclones may be selected during the initial response, whereas surviving cells can acquire additional alterations or adopt transient phenotypes that reduce immune recognition. Treatment-induced tissue injury may further reorganize stromal, vascular and myeloid compartments into protective niches, preserving residual populations with reduced visibility, impaired susceptibility to immune killing or limited exposure to immune effector cells. For clarity, these mechanisms can be broadly categorized as cell-intrinsic modes arising within malignant cells and cell-extrinsic modes mediated by immune, stromal, vascular and metabolic components of the tumour microenvironment, although the two modes are interconnected through bidirectional signalling. Viewed temporally, pre-existing tumour-intrinsic or microenvironmental barriers predominantly underlie primary resistance. Treatment-induced and potentially reversible changes, including stem-like plasticity, epigenetic immune concealment and niche remodelling, contribute mainly to adaptive resistance during therapeutic immune activation, the selective bottleneck and residual equilibrium. By contrast, selection or stabilization of antigen loss, signalling defects and suppressive niches can establish acquired resistance and drive secondary immune escape, although individual mechanisms may operate across more than one stage (17).

### 3.1 Cell-intrinsic genetic selection reduces tumour immune visibility

Genetic evolution provides one of the most durable routes to therapy-associated immune escape. Antitumour immunity preferentially eliminates cells that express recognizable antigens, retain functional antigen-presentation machinery and remain responsive to immune effector signals (30). This selective pressure can therefore enrich tumour populations carrying alterations that reduce antigenicity or interrupt immune recognition (31). Such alterations may be present at low frequency before treatment or emerge during prolonged therapy, but both scenarios can produce a similar clinical phenotype of secondary resistance.

Loss or downregulation of tumour antigens is particularly relevant when treatment depends on recognition of defined targets. Immune checkpoint blockade may select for clones with reduced neoantigen expression, whereas adoptive cellular therapies and bispecific immune engagers can impose even stronger pressure on cells expressing a single dominant antigen (32). Antigen-negative or antigen-low populations can subsequently expand despite continued immune activation (33). Defects in antigen processing and presentation provide another route of escape. Alterations involving HLA molecules, β2-microglobulin, transporters associated with antigen processing or components of the immunoproteasome can reduce peptide presentation at the tumour-cell surface and thereby impair CD8^+^ T-cell recognition. These mechanisms are well established within the classical immunoediting framework and can also be selected during treatment.

Tumour cells may additionally escape by becoming insensitive to inflammatory and cytotoxic signals. Alterations in IFN–JAK–STAT signalling can reduce induction of antigen-presentation genes, chemokines and growth-inhibitory programmes (24). Mutations or loss of function in JAK1, JAK2 or related pathway components may therefore simultaneously decrease tumour visibility and weaken the direct antitumour effects of interferons (34). Resistance can also arise downstream of immune recognition through altered apoptotic signalling, enhanced DNA damage repair or increased expression of survival factors (35). In these settings, tumour-specific T cells may still recognize malignant cells but fail to eliminate them efficiently.

Importantly, genetic escape is not always complete or uniform. Different metastatic sites or tumour regions may contain distinct antigen-presentation defects, and only a subset of cells may carry a particular resistant alteration (36). Treatment can therefore reduce overall tumour burden while leaving behind genetically restricted reservoirs that later repopulate the tumour. This spatial and clonal heterogeneity limits the value of single-site baseline biopsies and supports longitudinal analysis of residual and progressing lesions (37).

### 3.2 Therapy-resistant stem-like cancer cells create reversible immune-escape states

Not all therapy-associated immune escape is encoded by stable genetic change. A particularly important component of this non-genetic response is the emergence or enrichment of therapy-resistant stem-like cancer cells. Rather than necessarily representing a fixed and rare cellular hierarchy, stemness may also exist as a reversible functional state induced or stabilized by therapeutic and immune pressure. These cells can combine self-renewal capacity with quiescence, enhanced stress tolerance and lineage plasticity, while coordinating epigenetic reprogramming, metabolic adaptation and active immunoregulatory signalling. Together, these properties reduce their susceptibility to therapy- and immune-mediated elimination, thereby providing a reservoir from which residual disease and secondary immune escape may emerge (38). Because stem-like states can arise rapidly and affect substantial fractions of a tumour population, they may support survival during the therapeutic bottleneck before genetically fixed resistance becomes established.

Epigenetic silencing can reduce the expression of tumour antigens, HLA molecules, chemokines and interferon-responsive genes without altering their DNA sequence. Such suppression may allow tumour cells to remain temporarily invisible during periods of intense immune pressure. Similarly, dedifferentiation or lineage switching can alter the antigenic programme of malignant cells and make previously recognized targets less abundant or absent (39). This mechanism is particularly relevant to therapies directed against lineage-associated antigens, but it can also influence responses to checkpoint blockade by changing tumour immunogenicity and cytokine production.

In therapy-resistant stem-like populations, epigenetic regulation can coordinate the maintenance of stemness with reversible immune concealment. DNA methylation and histone-modification programmes may suppress lineage-associated antigens, HLA class I molecules, components of the antigen-processing machinery and interferon-responsive genes without requiring irreversible genetic loss. For example, epigenetic silencing of transporter associated with antigen processing 1 (TAP1) has been demonstrated in Aldefluor-positive breast cancer stem cells and was associated with impaired antigen processing and enhanced survival under immune pressure (40). Such epigenetically maintained states are particularly relevant to therapy-induced immunoediting because they can arise rapidly, preserve the underlying antigen-presentation genes and potentially be reversed by differentiation or epigenetic therapies.

Treatment can also induce stress-associated, quiescent or persister-like states. Cells in these states may proliferate slowly, reduce biosynthetic activity and become less susceptible to both cytotoxic drugs and immune-mediated killing. Some may retain the capacity to re-enter the cell cycle once treatment pressure is reduced, providing a cellular reservoir for relapse (41). Epithelial–mesenchymal plasticity and related transcriptional programmes can further promote immune escape by altering antigen presentation, death-receptor signalling and interactions with stromal cells (42).

These properties position therapy-resistant stem-like cells at the intersection between conventional treatment resistance and immune escape. While therapy preferentially removes differentiated and immune-visible tumour cells, surviving stem-like populations may display reduced or altered lineage-antigen expression, increased inhibitory checkpoint signalling and enhanced resistance to apoptosis or other immune-effector mechanisms. Epithelial–mesenchymal transition can further enrich PD-L1 in stem-like cancer cells through the β-catenin–STT3 axis, thereby reducing their susceptibility to immune-cell-mediated killing. Following treatment withdrawal or adaptation, these surviving cells may re-enter the cell cycle and regenerate phenotypically heterogeneous tumours, including both stem-like and more differentiated progeny. Therapy-resistant stem-like cells can therefore function as a persistent cellular reservoir that connects the selective bottleneck of treatment with residual equilibrium, immunotherapy resistance and subsequent relapse (43).

Metabolic adaptation and immune regulation further reinforce the persistence of therapy-resistant stem-like cells. These cells can adjust glycolysis, oxidative phosphorylation and antioxidant programmes to survive nutrient limitation and treatment-induced stress, while also releasing factors that remodel neighbouring tumour and immune cells. For example, breast cancer stem cell-derived macrophage migration inhibitory factor (MIF) promotes glycolytic reprogramming in surrounding tumour cells and contributes to an immunosuppressive microenvironment, thereby linking stemness-associated metabolism with immune evasion. Such effects allow stem-like populations to protect both themselves and more differentiated tumour cells, prolonging residual equilibrium and increasing the likelihood of subsequent immune-resistant relapse (44).

Together, these findings indicate that therapy-resistant stem-like cells integrate three interdependent forms of non-genetic immune escape: epigenetic suppression of tumour visibility, metabolic adaptation that supports survival and immunosuppression, and active remodelling of the surrounding immune compartment. The distinction between genetic and non-genetic escape has direct therapeutic implications. Stable loss of β2-microglobulin or a target antigen may require alternative immune effectors that do not depend on conventional HLA-restricted recognition. By contrast, reversible antigen suppression or epigenetic silencing may be amenable to differentiation therapy, epigenetic modulation or restoration of interferon responsiveness. However, these categories are not mutually exclusive. Prolonged residence in a reversible escape state may create time for the accumulation of stable mutations, allowing temporary adaptation to evolve into fixed resistance (45).

### 3.3 Cell-extrinsic ecosystem remodelling protects residual tumour cells

Immune escape can also occur without major alterations in the tumour cell itself. Residual malignant cells may survive because they occupy tissue regions that limit immune-cell access or function. These spatially protected niches are shaped by cancer-associated fibroblasts, extracellular matrix, abnormal vasculature, hypoxia and suppressive myeloid populations (46). Therapy can disrupt these compartments, but it can also stimulate wound-healing and tissue-repair responses that reinforce local protection.

Fibroblast activation and extracellular matrix deposition can physically restrict T-cell movement and retain lymphocytes in peritumoural stromal regions. TGF-β-associated programmes may further suppress inflammatory chemokines and promote immune exclusion (47). Abnormal tumour vessels can reduce perfusion, adhesion-molecule expression and transendothelial lymphocyte migration, while hypoxia and nutrient deprivation impair T-cell effector function (48). Metabolites such as adenosine, lactate and kynurenine can create localized immunosuppressive conditions even when systemic immune activation is present (49).

Myeloid cells are central organizers of these protective ecosystems. Tumour-associated macrophages, myeloid-derived suppressor cells and certain neutrophil states can inhibit T-cell activity, remove inflammatory signals and support tissue repair after treatment (50). Their accumulation may be promoted by chemotherapy- or radiotherapy-induced injury, creating a transition from early immune activation to later immunosuppression. Regulatory T cells and dysfunctional lymphocyte states can further stabilize this environment (51). Spatial heterogeneity means that elimination, equilibrium and escape may coexist within the same tumour. One region may contain active T-cell-mediated killing, another may harbour dormant residual cells, and a third may already support expanding immune-evasive clones (52). Bulk molecular measurements can obscure these local differences. Spatial profiling and serial sampling are therefore essential for identifying the small residual niches that ultimately drive relapse (53).

Together, genetic selection, non-genetic plasticity and spatial ecosystem remodelling form an interconnected escape network. Genetic defects can reduce immune recognition, plastic states can provide rapid and reversible protection, and suppressive niches can sustain the surviving populations long enough for secondary resistance to mature. Effective intervention will therefore require not only identifying which mechanism is present, but also determining when it emerges and how it changes across the treatment continuum. Importantly, tumour-cell-intrinsic programmes can remodel the surrounding immune ecosystem, whereas immune- and stromal-derived signals can reinforce tumour-cell plasticity and resistance, creating bidirectional feedback between the two modes.

## 4 Pharmacological interception of therapy-induced immunoediting

The therapeutic relevance of the immunoediting continuum depends on whether evolving escape states can be identified and intercepted pharmacologically. A simple strategy of adding further immune stimulation is unlikely to be effective across all stages because residual tumours may persist through fundamentally different mechanisms (54). Accordingly, the therapeutic approaches discussed below can be viewed as strategies to overcome pre-existing barriers, reverse treatment-induced and potentially reversible escape states, disrupt cell-extrinsic protective niches or bypass genetically fixed acquired resistance. Reversible suppression of antigen presentation may remain pharmacologically tractable, whereas irreversible loss of β2-microglobulin or a dominant target antigen may require alternative recognition pathways. Likewise, a tumour that remains visible but spatially inaccessible requires a different intervention from one in which tumour-reactive lymphocytes have become dysfunctional. Pharmacological interception should therefore be directed towards the dominant limitation in tumour visibility, immune-cell access or effector susceptibility and should ideally be deployed before resistant populations become clonally fixed (55).

### 4.1 Restoring tumour visibility and immune sensitivity

Epigenetic therapies provide one approach to reversing non-genetic immune concealment. DNA methyltransferase inhibitors such as azacitidine and decitabine, and histone deacetylase inhibitors such as entinostat, can increase the expression of cancer–testis antigens, components of the antigen-processing machinery and interferon-responsive genes in preclinical models. These effects provide a rationale for combining epigenetic modulation with PD-1 or PD-L1 blockade, particularly when immune escape reflects reversible transcriptional suppression rather than structural loss of the presentation machinery. In the phase II ENCORE 601 study, entinostat plus pembrolizumab was evaluated in patients with PD-(L)1-resistant or refractory non-small-cell lung cancer. Clinical activity was limited to a subgroup of patients, illustrating both the potential and the limitations of attempting to pharmacologically restore immunogenicity in an unselected resistant population (56). In metastatic uveal melanoma, the phase II PEMDAC study reported an objective response rate of 14% with entinostat plus pembrolizumab, but the modest efficacy again indicated that epigenetic priming is not sufficient when additional tumour-intrinsic or microenvironmental barriers remain dominant (57).

These findings emphasize the need to distinguish reversible immune concealment from genetically fixed escape. Epigenetic agents cannot be expected to restore conventional CD8^+^ T-cell recognition when B2M is completely lost or when essential HLA alleles have been deleted. Such tumours may instead require HLA-independent approaches, including natural killer-cell-based therapy, bispecific or multispecific immune engagers, or recognition of an alternative tumour antigen. Pharmacological restoration of tumour visibility should therefore be linked to biomarkers confirming that the relevant antigen-presentation pathway remains structurally intact.

### 4.2 Disrupting spatial and metabolic protection

Tumour cells can remain antigenic and sensitive to killing yet survive because vascular, stromal or metabolic barriers prevent effective immune-cell access. The combination of atezolizumab and bevacizumab provides an established clinical example of simultaneously targeting immune inhibition and a vascular–immunosuppressive pathway. In the phase III IMbrave150 trial, atezolizumab plus bevacizumab significantly improved overall and progression-free survival compared with sorafenib in patients with unresectable hepatocellular carcinoma (58). Although the clinical benefit cannot be attributed exclusively to vascular normalization, inhibition of VEGF can improve vascular function, reduce VEGF-associated immune suppression and facilitate immune-cell trafficking, thereby addressing several components of spatial immune escape. This regimen demonstrates that targeting the tissue context in which immune effectors operate may be as important as directly releasing inhibitory checkpoints.

Metabolic immune suppression represents another potentially actionable component of spatial protection. CD73 converts extracellular AMP to adenosine, which suppresses T-cell and natural killer-cell function in hypoxic tumour regions. The anti-CD73 antibody oleclumab has therefore been combined with the PD-L1 inhibitor durvalumab. In the phase II NeoCOAST platform trial in resectable non-small-cell lung cancer, durvalumab plus oleclumab produced a higher major pathological response rate than durvalumab alone and was associated with pharmacodynamic evidence of immune activation (59). Oleclumab remains investigational, and the relatively small non-comparative cohorts prevent definitive conclusions regarding clinical benefit. Nevertheless, the study illustrates how a drug combination can be designed to remove a metabolically defined layer of immune protection rather than merely intensifying checkpoint inhibition.

The failure of some mechanistically rational combinations is equally informative. Bintrafusp alfa was developed as a bifunctional molecule combining PD-L1 blockade with local sequestration of TGF-β, with the aim of simultaneously reversing T-cell inhibition and TGF-β-associated stromal exclusion. However, in a randomized phase III trial involving treatment-naive patients with PD-L1-high advanced non-small-cell lung cancer, bintrafusp alfa did not show superior efficacy to pembrolizumab (60). This result demonstrates that co-targeting two plausible escape pathways does not guarantee benefit when patients are not selected according to evidence that both pathways are functionally dominant. It also supports a central premise of therapy-induced immunoediting: combinations should be matched to the evolving escape state rather than selected solely on the basis of mechanistic plausibility.

### 4.3 Intercepting escape during residual equilibrium

The residual-equilibrium phase may provide the most favourable opportunity for pharmacological interception because tumour burden is low but surviving populations have not yet expanded into overt clinical relapse. The phase III IMvigor011 trial provides direct support for biomarker-guided treatment during this interval. Patients with high-risk muscle-invasive bladder cancer underwent serial circulating tumour DNA monitoring after cystectomy, and those who became ctDNA-positive were randomly assigned to atezolizumab or placebo. CtDNA-guided atezolizumab significantly prolonged disease-free and overall survival, whereas patients who remained persistently ctDNA-negative had favourable outcomes without immediate treatment escalation (61).

CtDNA positivity does not by itself establish that residual disease is maintained in an immunological equilibrium, nor does it identify the specific mechanism of immune escape. Nevertheless, IMvigor011 demonstrates that molecularly detectable residual disease can define a clinically actionable interval before radiographic recurrence. It also provides a practical model for adaptive therapy in which longitudinal biomarkers determine not only who should receive treatment, but also when pharmacological intervention should begin. Earlier exploratory analyses of postoperative urothelial cancer similarly showed that ctDNA-positive patients appeared more likely to benefit from adjuvant atezolizumab, whereas relapse despite treatment was associated with angiogenesis and fibroblast TGF-β signatures (62). These observations suggest that molecular residual disease may need to be combined with immune and spatial biomarkers to select the most appropriate intervention.

These clinical examples support a stage- and mechanism-matched approach to therapy-induced immunoediting rather than a simple escalation of immune stimulation. Reversible immune concealment may be approached through epigenetic modulation, spatial or metabolic protection may require vascular, stromal or adenosine-pathway intervention, and antigen loss or HLA-dependent recognition failure may require alternative immune-recognition strategies. Longitudinal ctDNA surveillance may further identify residual disease before secondary escape becomes clinically established. Importantly, negative or modestly active combinations are also informative, because they reveal the limitations of mechanistic plausibility in the absence of biomarker-guided patient selection and appropriate timing (**Table 1**). Therefore, translating therapy-induced immunoediting into treatment design requires identifying the dominant escape state, assessing whether it remains reversible and selecting interventions at a stage when tumour evolution can still be redirected.

**Table 1:** Representative pharmacological strategies across the therapy-induced immunoediting continuum.

Escape mechanism or stage	Drug or regimen	Pharmacological rationale	Tumour context and treatment setting	Clinical status	Relevance to immunoediting	Refs
Acquired immune invisibility	Pembrolizumab or nivolumab	PD-1 blockade restores T-cell activity	Metastatic melanoma; acquired resistance after PD-1 blockade	Approved; acquired JAK1/JAK2 or B2M defects reported after response	Demonstrates selection of IFN-insensitive or MHC-I-deficient clones	(23)
Compensatory T-cell inhibition	Nivolumab plus relatlimab	Dual PD-1 and LAG-3 blockade	Previously untreated advanced melanoma	Approved in advanced melanoma; RELATIVITY-047	Intercepts alternative checkpoint-mediated escape	(63)
VEGF-associated immune exclusion	Atezolizumab plus bevacizumab	PD-L1 blockade plus VEGF inhibition	Unresectable HCC; first-line treatment	Approved in unresectable HCC; IMbrave150	Targets immune suppression and vascular barriers simultaneously	(64)
Adenosine-mediated suppression	Durvalumab plus oleclumab	PD-L1 and CD73 blockade	Resectable or stage III NSCLC; neoadjuvant or consolidation treatment	Investigational; COAST/NeoCOAST	Reduces adenosine-dependent protection of residual tumour cells	(59, 65)
NKG2A-mediated inhibition	Durvalumab plus monalizumab	Blocks PD-L1 and inhibitory NKG2A signalling	Stage III NSCLC; post-chemoradiotherapy consolidation	Investigational; COAST.	Restores NK- and CD8^+^ T-cell effector activity	(65)
Epigenetic immune concealment	Entinostat plus pembrolizumab	HDAC inhibition may restore antigen presentation and IFN responses	Anti-PD-(L)1-resistant metastatic NSCLC	Phase II; limited activity in resistant NSCLC	Suggests benefit may require reversible epigenetic escape and biomarker selection	(56)
TGF-β-associated exclusion	Bintrafusp alfa	Combined PD-L1 blockade and TGF-β trapping	Treatment-naive advanced NSCLC	Phase III negative versus pembrolizumab	Shows that mechanistic plausibility alone does not ensure clinical benefit	(60)
Insufficient effector expansion	Bempegaldesleukin plus nivolumab	IL-2 pathway stimulation plus PD-1 blockade	Previously untreated advanced melanoma	Phase III negative in melanoma	Nonspecific immune amplification may not overcome dominant escape mechanisms	(66)
Defective immune priming	T-VEC plus pembrolizumab	Oncolysis and antigen release plus PD-1 blockade	Unresectable advanced melanoma	Phase III negative; MASTERKEY-265	Antigen release alone may be insufficient when downstream barriers persist	(67)
Single-antigen escape	CD19 CAR-T therapies	HLA-independent recognition of CD19	Relapsed/refractory aggressive B-cell lymphoma	Approved in B-cell malignancies; CD19-low or negative relapse reported	Provides a clear model of treatment-driven antigen loss	(68)
Prevention of antigen escape	Dual CD19/CD22 CAR-T	Simultaneous targeting of two antigens	Relapsed/refractory B-cell malignancies	Early-phase clinical studies	Reduces but does not eliminate antigen-dependent escape	(69)
Alternative target recognition	CD22 CAR-T	Targets CD22 after CD19-directed failure	Large B-cell lymphoma after CD19 CAR-T failure	Investigational after CD19 CAR-T relapse	Demonstrates target switching after therapy-induced antigen editing	(70)
HLA-independent T-cell redirection	Glofitamab or epcoritamab	CD20×CD3 bispecific antibodies recruit endogenous T cells	Relapsed/refractory large B-cell lymphoma	Approved in relapsed or refractory B-cell lymphoma	Provides an alternative recognition route after CD19-directed selection	(71, 72)
Molecular residual disease	ctDNA-guided atezolizumab	Treats molecular relapse before radiographic progression	Post-cystectomy molecular residual disease in bladder cancer	Positive phase III IMvigor011	Supports residual equilibrium as an actionable treatment window	(61)

## 5 Residual equilibrium and longitudinal tracking of evolving immune escape

Residual disease should not be regarded simply as incomplete tumour regression. Within the therapy-induced immunoediting continuum, it may represent a dynamic equilibrium in which surviving malignant cells remain constrained by ongoing treatment and host immunity but have not been fully eliminated (5). Clinically, this state may correspond to pathological residual disease, molecular residual disease, radiographically occult persistence, stable disease or a partial response. The balance is inherently unstable: continued immune surveillance may maintain long-term control, whereas further adaptation of residual cells or deterioration of immune function can shift the system towards secondary escape and overt relapse. Importantly, molecular or pathological residual disease does not automatically prove the existence of an immunological equilibrium, but it identifies a period in which tumour burden is low and evolutionary selection remains active.

The residual population is unlikely to reproduce the biological composition of the pretreatment tumour. Initial therapy preferentially removes sensitive and immune-visible cells, thereby enriching surviving populations with reduced antigen expression, impaired interferon responsiveness, altered apoptotic sensitivity, reversible persister-like states or dependence on protective stromal and myeloid niches (73). These cells may remain clinically undetectable while continuing to evolve under immune pressure (74). Residual disease therefore functions both as a reservoir for later relapse and as a potentially favourable window for interception before resistant clones expand, diversify and become more difficult to control. The pharmacological strategies discussed above are most likely to succeed when they are applied according to the dominant mechanism of persistence rather than tumour burden alone.

Studying this interval requires experimental models that reproduce treatment response, residual survival and subsequent escape over time. Conventional tumour cell lines, endpoint animal studies and single pretreatment biopsies can identify candidate resistance mechanisms, but they cannot reliably distinguish pre-existing resistant clones from states that are selected, induced or stabilized during therapy. Informative models should preserve four key features: tumour heterogeneity, sustained immune selection pressure, spatial interactions within the tumour microenvironment and longitudinal sampling. Immunocompetent syngeneic and genetically engineered models can capture immune-mediated selection in an intact host, whereas serially treated tumour models can generate matched sensitive, residual and relapsing states (75). Patient-derived organoids and explants may retain tumour heterogeneity, but their value for studying immunoediting depends on the inclusion of autologous lymphoid, myeloid and stromal components. Tumour–immune co-cultures, microfluidic platforms and organ-on-chip systems can further model antigen-specific killing, immune-cell migration and vascular or stromal barriers under controlled conditions. Humanized mouse models provide an additional translational bridge, although incomplete immune reconstitution and donor variability remain important limitations (76, 77).

Biomarker development should likewise move beyond static prediction of response. Baseline biomarkers such as tumour mutational burden, antigen-presentation capacity, inflammatory gene signatures and spatial immune-cell distribution may estimate initial treatment susceptibility, but they do not reveal how the tumour is changing. Early pharmacodynamic biomarkers should determine whether therapy has activated the intended immune pathway, expanded tumour-reactive lymphocytes or increased tumour-cell killing (78). During residual equilibrium, the priority is to identify surviving populations and emerging escape states, including antigen loss, impaired HLA or interferon signalling, lineage plasticity, persistent suppressive myeloid programmes and spatial immune exclusion. At progression, analysis should establish whether relapse reflects expansion of a pre-existing resistant clone, acquisition of a new genetic alteration or stabilization of a previously reversible cell state.

No single specimen can fully capture this process. Molecular monitoring may be complemented by longitudinal assessment of skeletal muscle status, muscle strength, cardiorespiratory fitness and patient-reported function. Although these measures do not directly identify immune escape, they can indicate whether patients retain sufficient functional reserve to tolerate maintenance, combination or salvage therapy. The most informative framework combines pretreatment, early on-treatment, residual-disease and progression samples. Tissue analysis can be complemented by circulating tumour DNA to track clonal persistence and molecular residual disease, while longitudinal T-cell receptor profiling may reveal expansion, contraction or replacement of tumour-reactive lymphocyte populations (79). Single-cell and spatial profiling can identify rare residual states and determine whether immune effectors remain excluded, dysfunctional or physically separated from surviving tumour cells (80). Integrating these measurements may allow biomarkers to function not only as predictors of response, but also as indicators of state transition across the immunoediting continuum.

The central clinical challenge is to distinguish residual tumours that remain under effective immune control from those already moving towards secondary escape. Tumour size alone cannot resolve this distinction. A longitudinal assessment of tumour-cell state, clonal dynamics, immune pressure and spatial organization is therefore required. Such an approach could support adaptive treatment strategies in which therapy is modified according to the evolving mechanism of persistence, rather than continued unchanged until radiographic progression confirms that immune escape is already established.

## 6 Discussion and future directions

Therapy-induced immunoediting provides a dynamic framework for understanding why initially effective anticancer treatments may ultimately culminate in immune escape. Rather than representing a single resistance mechanism, relapse can emerge through the sequential selection of genetically altered clones, therapy-resistant stem-like or persister states and spatially protected residual niches. Therapy-resistant stem-like states are particularly relevant to this continuum because they integrate epigenetic immune concealment, metabolic adaptation and active regulation of the immune microenvironment, enabling residual tumour cells to survive immune pressure and retain the capacity to regenerate heterogeneous disease. This perspective highlights the limitations of static baseline biomarkers and supports longitudinal assessment across pretreatment, early response, residual disease and progression. Future studies should integrate serial tissue sampling, circulating tumour DNA, T-cell receptor profiling and spatial or single-cell analyses to distinguish pre-existing resistance from treatment-selected or newly acquired escape.

Clinical translation should consider tumour type, disease stage and the dominant resistance mechanism. In B-cell malignancies with defined surface antigens, CAR-T cells or bispecific antibodies may provide effective HLA-independent recognition, whereas antigenic heterogeneity or target loss may favour dual- or alternative-antigen strategies. In solid tumours with preserved antigen presentation and immune infiltration, checkpoint-based combinations may be appropriate, while immune-excluded tumours dominated by abnormal vasculature, suppressive stroma or metabolic barriers may require VEGF-, TGF-β- or adenosine-directed approaches. Reversible epigenetic concealment may support epigenetic priming, whereas fixed B2M or HLA loss requires alternative recognition pathways. Treatment timing is also important: molecular residual disease or residual equilibrium may offer an opportunity for early mechanism-matched intervention before heterogeneous resistant populations become established.

A major translational challenge is to match pharmacological intervention to the dominant and potentially reversible escape mechanism. Clinical experience with VEGF inhibition, epigenetic modulators, adenosine-pathway blockade, dual checkpoint inhibition and antigen-directed cellular therapies shows that mechanistic plausibility alone is insufficient without biomarker-guided patient selection and appropriate timing. Residual equilibrium may represent the most actionable intervention window, when tumour burden remains low but resistant populations have not yet undergone extensive expansion. Within this adaptive-care framework, rehabilitation may help preserve muscle function, cardiorespiratory capacity and treatment tolerance, thereby maintaining patients’ ability to receive stage-matched interventions during residual disease. Ultimately, adaptive treatment strategies should aim not simply to intensify immune activation, but to identify when, where and through which route immune escape is emerging and to intervene before secondary resistance becomes clinically established.
